# WHO guidelines on physical activity, sedentary behaviour, and sleep for children under 5: a qualitative study of Mongolian stakeholder perceptions

**DOI:** 10.1093/heapol/czag037

**Published:** 2026-03-17

**Authors:** Ankhmaa Byambaa, Rachel A Jones, Anthony D Okely

**Affiliations:** Nutrition Department, National Centre for Public Health, 17 Peace Avenue, Ulaanbaatar 13381, Mongolia; School of Medical, Indigenous and Health Sciences, Faculty of Science, Medicine and Health, University of Wollongong, Northfields Avenue, Wollongong, NSW 2522, Australia; School of Education, Faculty of Arts, Society and Business, University of Wollongong, Northfields Avenue, Wollongong, NSW 2522, Australia; School of Medical, Indigenous and Health Sciences, Faculty of Science, Medicine and Health, University of Wollongong, Northfields Avenue, Wollongong, NSW 2522, Australia

**Keywords:** early childhood, guidelines, movement behaviours, implementation barriers, LMIC

## Abstract

WHO’s ‘24-hour movement guidelines’ provide integrated recommendations for physical activity, sedentary behaviour, and sleep to improve health outcomes in children aged 0–5. This study aimed to examine stakeholders’ perceptions about these guidelines to inform its future adoption, adaptation, and implementation decisions in Mongolia. Key stakeholders involved in young children's health and development were recruited through purposive sampling and snowball methods. Semi-structured interviews spanning 30–50 minutes were conducted with 38 participants using an interview guide in Mongolian language: caregivers (*n* = 19), kindergarten teachers (*n* = 14), health and policy professionals (*n* = 5). Audio-recorded data were transcribed, translated to English, and thematically analysed using deductive thematic analysis. Four main themes were identified: (i) awareness and appropriateness, (ii) understanding of the guidelines, (iii) perceived factors influencing adoption and implementation, and (iv) dissemination and implementation strategies. Stakeholders were mostly unaware of the WHO guidelines but perceived them as useful, feasible and culturally appropriate. Stakeholders identified barriers including competing priorities (e.g. work/family obligations), environmental challenges (lack of child-friendly spaces, cold weather, air pollution), and knowledge and capacity gaps. Stakeholder-recommended strategies for effective dissemination and implementation were kindergarten-based integration, training for caregivers and teachers, and adequate resource provision. Dissemination should use multichannel communication approaches emphasizing benefits, potential consequences of non-adherence, and provide specific, culturally relevant examples. This study provides first examination of stakeholder perspectives on WHO 24-hour movement guidelines in Central Asia. These preliminary findings suggest that successful adoption may require cultural adaptation, stakeholder education, and leveraging existing infrastructure and policy frameworks, though education sector consultation would be needed for comprehensive feasibility assessment.

Key messages

- First study in Mongolia reveals strong stakeholder support for WHO 24-hour movement guidelines for early years despite near-universal unawareness.
- Movement behaviours are systematically deprioritized in favour of cognitive development and academic preparation across kindergarten curricula, family priorities, and policy levels.
- Grandparents should be targeted as primary stakeholders due to their central caregiving role in multigenerational Mongolian families.
- Kindergarten-centred implementation leveraging existing infrastructure emerged as stakeholder’s preferred approach, with participants recommending integration into established education and health systems.

## Introduction

In response to rising rates of physical inactivity, the World Health Organization (WHO) developed ‘Guidelines for Physical Activity, Sedentary Behaviour, and Sleep for children under the age of five years’ (World Health Organization 2019b). The WHO guidelines consider physical activity, sedentary behaviour, and sleep as interrelated behaviours that collectively make up a full 24-hour day and are referred to as ‘24-hour movement behaviours’. These integrated guidelines are based on evidence that meeting recommended durations for each behaviour collectively supports children’s healthy growth and development (World Health Organization 2019b, Willumsen and Bull 2020).

While research demonstrates significant health benefits for children who adhere to these guidelines, global compliance remains concerningly low (Kuzik et al. 2017, Rollo et al. 2020, Chong et al. 2024). For example, a pooled analysis of movement behaviour data from 33 countries reported that only 14.3% of children comply with all recommendations. Variations exist between high-income (14.4%), upper-middle income (11.9%), and low and lower-middle income countries (16.6%), as well as between geographic regions (highest adherence in Africa—23.9%; and lowest in North and South America—7.7%) (Chong et al. 2024). Irrespective of region, levels of compliance are far below optimal, highlighting the need for country-specific approaches to improve guideline adherence worldwide. Further, this is critical as many low- and middle income countries (LMICs) grapple with considerable health challenges posed by rapid urbanization and shifts towards more sedentary lifestyle.

Mongolia represents an example, lacking any guidelines on children’s movement behaviours despite concerning health statistics. A study by Norov et al. (2023) of childhood overweight and obesity rates in Mongolia, Indonesia, and Vietnam revealed growing rates of overweight and obesity in all three countries, with Mongolia having ‘high’ prevalence among children under five. In addition, only 7% of Mongolian preschoolers adhere to the all recommendations of the WHO 24-hour movement guidelines (Byambaa et al. 2024). Together, the lack of guidelines, rising obesity rates, and poor adherence to global recommendations demonstrate the need for Mongolian-specific 24-hour movement guidelines for children.

However, the development of country-specific movement guidelines is costly and demanding, which are often too extensive for LMICs (Schünemann et al. 2017). Further, the development of country-specific guidelines requires substantial resources, expertise, and rigorous systematic review of existing research (Schünemann et al. 2017, World Health Organization 2023). Therefore, adopting and locally adapting established global guidelines may offer a more cost-effective and feasible approach.

There are a number of examples of countries that have adopted and adapted the WHO 24-hour movement guidelines, but most have primarily been in high-income settings such as Hong Kong and Singapore (Capio et al. 2022, Loo et al. 2023). South Africa released the first LMIC-specific ‘24-hour movement guidelines for birth to 5 years’ in 2018, using the GRADE-ADOLOPMENT approach based on Australian 24-hour integrated guidelines for the early years and the WHO guidelines (Draper et al. 2020). Many studies from LMICs have focused primarily on compliance assessment, rather than undertaking the more complex but crucial process of guideline adaptation for their specific contexts. The adaptation process represents a vital step in moving from global recommendations to implementable national policies (World Health Organization 2023). Guidelines are most likely to be effective when contextualized rather than simply translated, as they need to align with local resources, health system structures, and needs and values of target-population (World Health Organization 2023). For example, it is essential to consider Mongolia’s extreme climate condition, traditional nomadic culture, urban-rural disparities, and Soviet-era institutional structures, in the contextual adaptation of the WHO guidelines for Mongolian settings.

Successful guideline adaptation and implementation depends on understanding and addressing the perspectives and concerns of stakeholders who will be using the guidelines (World Health Organization 2023). Caregivers, teachers, and health and policy professionals are key stakeholders that influence children’s lifestyle and behaviours, making them valuable sources for ensuring that adapted guidelines are relevant, acceptable and feasible within the local context. Previous studies exploring stakeholders’ perceptions of movement guidelines across various countries, including studies of WHO 24-hour movement guidelines and earlier national guidelines from Canada and Australia, have revealed generally positive attitudes among parents and teachers, who recognize their importance for children’s health (Faulkner et al. 2016, Stanley et al. 2020, Capio et al. 2022, Draper et al. 2022). Common implementation barriers from these studies included low awareness about the guidelines, parental lack of time, insufficient curriculum time, limited physical spaces, and difficulties in quantifying children’s physical activity levels (Capio et al. 2022, Draper et al. 2022, Capio et al. 2025). These findings highlight the importance of careful messaging strategies to avoid parental guilt and stress when children do not meet recommendations. Further these studies have identified various context-specific dissemination approaches that are needed for successful guideline uptake and wide dissemination (Faulkner et al. 2016, Stanley et al. 2020, Capio et al. 2022). These findings demonstrate the importance of exploring stakeholder perspectives for effective guidelines adaptation and implementation, particularly when adopting global recommendations to align with local context, needs, and resources.

While numerous countries conducted studies to measure compliance with the WHO guidelines, only a limited number (i.e. 4) have explored stakeholder perspectives about the guidelines. Without understanding barriers and facilitators, guidelines remain poorly implemented, perpetuating the low compliance rates. Moreover, no such research has been conducted in a LMIC within the Central Asian region. Therefore, we aimed to investigate Mongolian key stakeholders’ perceptions about the WHO 24-hour movement guidelines exploring their awareness of the guidelines, cultural appropriateness, perceived barriers, and implementation preferences to inform the potential adoption, adaptation, and implementation in Mongolia.

## Methods

We followed the consolidated criteria for reporting qualitative research (COREQ) 32-item checklist to report the findings (Tong et al. 2007) and included the checklist as a Supplementary File 3.

### Reflexivity

The research team comprises the first author (A.B.), a female PhD student of Mongolian nationality studying at an Australian tertiary university, and two senior researchers of Australian nationality (R.A.J. and A.D.O.) with expertise in physical activity and early childhood health. A.B. holds a substantive position as a research officer at the National Centre for Public Health in Mongolia under the Ministry of Health, a leading research institute responsible for providing research evidence for health policymaking in Mongolia. This position enabled initial contact with professional public health networks used in recruitment. A.B.’s dual position (i.e. Mongolian national with established professional network and knowledge of local context and researcher trained in Western methodological research practices) shaped both data collection and interpretation. Authors R.A.J. (female) and A.D.O. (male), both hold doctoral degrees and academic positions at an Australian tertiary university. R.A.J. and A.D.O. were previously involved in developing the Australian 24-hour movement guidelines for early years and conducted similar research examining Australian stakeholder perceptions, providing extensive experience in guideline development and implementation research.

The research team held prior assumptions that the WHO 24-hour movement guidelines are important for child health and would likely require cultural adaptations for the Mongolian context, particularly regarding the stakeholder interpretation and understanding. These assumptions were regularly discussed throughout the data collection and analytical process.

To address potential bias arising from professional relationships and preconceptions, the research team engaged in regular reflexive discussions during data collection and analysis, examining how positionality and assumptions might influence participant responses and interpretive decisions. The first author (A.B.) did a reflexive journaling to document analytical insights. The broader research team provided external perspective during analysis to balance assumptions and challenge interpretations. Participants were assured of confidentiality and informed that the research was conducted independently from A.B.’s institutional role.

### Design and sampling

A qualitative descriptive study design was employed to explore stakeholders’ perceptions of the WHO 24-hour movement guidelines. The selection of participants for this study was based on the socioecological model (SEM), a comprehensive framework that recognizes the complex interplay of factors influencing children’s health and behaviours (Davison & Birch 2001, Sallis et al. 2006). This model posits that a child’s health-related behaviours are shaped by multiple levels of influence: (i) individual level: child’s own characteristics; (ii) interpersonal level: parental and family characteristics; (iii) community level: community, societal and demographic characteristics; (iv) policy and systems level: supportive policies, legal frameworks, and services. By selecting participants from various levels of this model, we aimed to capture a holistic understanding of the factors that may influence the adoption, adaptation, and implementation of the WHO guidelines in Mongolia. Three key stakeholder groups were selected: parents or caregivers from diverse socioeconomic backgrounds (interpersonal level of the SEM), early childhood educators and teachers who work at urban/rural and private/public kindergartens (community level of the SEM), health professionals, researchers, and policy professionals from public health sector (community and policy/systems level of the SEM).

Participants were recruited using purposeful sampling and snowball sampling methods (Palinkas et al. 2015). The research team had previously conducted the Mongolian component of the ‘SUNRISE International Study of Movement Behaviours in Early Years’ (Okely et al. 2021 ), providing access to an established network of kindergartens across multiple regions. Principals of these kindergartens were invited to participate in the current study via phone calls and emails. Principals were asked to distribute recruitment flyers within their kindergarten communities. Through this existing network, we recruited kindergarten teachers and parents of young children. Health professionals, policy makers, and public health researchers with expertise in child health, physical activity, or health promotion were identified through purposeful sampling, with additional participants recruited through professional networks and referrals.

The final sample comprised 38 participants. Sample adequacy was assessed through information power (Malterud et al. 2016) based on study characteristics including narrow aim, sample specificity, analytical approach, dialogue quality, and contextual specificity (see Data analysis section for more details). Participants were from three geographically distinct locations representing different levels of urbanization in Mongolia. Participants from Ulaanbaatar city were classified as urban, while those from the city’s outskirts in ger districts were classified as peri-urban. Participants from Tuv and Zavkhan provinces were classified as rural. Ulaanbaatar serves as the capital of Mongolia and primary urban centre, where almost half of the population lives. Ulaanbaatar’s central area has access to services, employment, and infrastructure, while the ger districts on the city’s periphery have more limited access to these amenities. Tuv province surrounds the capital city, and Zavkhan province represents a remote western region located ∼1000 km from Ulaanbaatar. This geographical representation aimed to capture diverse perspectives across Mongolia’s different development contexts. Characteristics of the three stakeholder groups are described below:

‘Caregivers’ (*n* = 19) included parents and grandparents of 0–5-year-old children, living in urban (*n* = 12), peri-urban (*n* = 2) and rural (*n* = 5) areas. This group comprised mothers, fathers, and grandparents, ensuring representation of the diverse primary caregiving roles.

‘Early childhood educators’ (*n* = 14) included kindergarten teachers and principals across urban (*n* = 8) and rural (*n* = 6) locations. All participants held professional qualifications in early childhood education or were currently studying early childhood education at relevant educational institutions. Their professional experience ranged from 2 to 30 years, providing insights from both early career and experienced educators familiar with young children’s developmental needs and educational practices.

‘Health and policy stakeholders’ (*n* = 5) comprised health workers (*n* = 2) and public health researchers (*n* = 3) involved in policy development related to nutrition, physical activity, health promotion, and non-communicable disease (NCD) prevention. This group provided perspectives on policy implementation feasibility and alignment with existing healthcare policy and initiatives.

All participants provided informed consent before participation. Written informed consent was obtained from participants in face-to-face interviews, while oral consent was obtained and digitally recorded for online interviews.

### Data collection

The first author (A.B.) conducted data collection through individual semi-structured interviews between January and March 2025. The interviews were arranged at convenient times for participants and were conducted face-to-face with participants at home (for caregivers) or at their workplace (for kindergarten teachers). Online interviews were conducted using Facebook messenger voice calls to accommodate participants from rural and remote locations. All interviews were conducted in Mongolian language, with average durations of 30 minutes for caregivers and teachers, and 50 minutes for health and policy professionals.

Prior to or at the beginning of the interview, participants received the WHO 24-hour movement guidelines in two versions: original English version and Mongolian-translated version (Supplementary File 1). The Mongolian-translated version of the guidelines was a near-direct translation of the official WHO technical document. As the guidelines have not yet been officially adopted in Mongolia, no changes were made to the wording or design. This approach was intended to prompt feedback on how the language and presentation could be improved for future adaptations tailored to the Mongolian context. Participants were given sufficient time to familiarize themselves with the content of the guidelines.

An interview guide was used for each participant group. Questions were adjusted to the participant’s background and expertise by using lay language for caregivers and teachers and more formal language for professionals. Discussions were focused on awareness of the WHO 24-hour movement guidelines (e.g. Before this study, were you aware of the WHO guidelines on 24-hour movement behaviours? ), perceived barriers to implementation (e.g. Can you think of any specific challenges that might make it hard for children to follow the recommendations? ) and potential strategies for dissemination and implementation (e.g. What kind of support or resources do you think would help you follow the guidelines and manage your child’s physical activity, sleep, and screen time better? ). The complete interview guides are provided as Supplementary File 2.

### Data recording and translation

All interviews were digitally audio recorded and transcribed verbatim using the Chimege software (Bolorsoft LLC, Mongolia). The first author (A.B.) reviewed each transcript for accuracy and made necessary corrections. Verified transcripts were then translated into English using Microsoft AI co-pilot, the AI tool officially recommended by and used at the University of Wollongong, Australia, with which all authors are affiliated. This platform complies with institutional data security and privacy standards, ensuring participant data confidentiality and protection. AI-assisted translation was used to improve efficiency, and the authors remained attentive to its limitations and ensured that all content was interpreted accurately through iterative checking. Translation into English prior to analysis was necessary as the coding process involved two researchers, one of whom was not proficient in Mongolian. We prioritized preserving participants’ intended meanings over strict literal equivalence (Abfalter et al. 2021). Therefore, English translations were meaning-driven rather than word-for-word literal translations, with some spoken-language expressions refined for clarity while maintaining original meaning. The first author (A.B.), bilingual in Mongolian and English, verified all AI-generated translations against original Mongolian transcripts to ensure semantic accuracy. The authors (A.B. and R.A.J.) carefully reviewed translations and conducted iterative discussions about translated terms and phrases to ensure shared understanding and preserve participants’ intended meanings. Interview transcripts were returned to participants for member checking and no corrections were received (Creswell and Miller 2000).

### Data analysis

Data were analysed thematically following Braun and Clarke’s approach (2006). We employed a deductive, question-driven analytical approach where our research questions provided the analytical framework (Braun and Clarke 2019). Data analysis followed the following stages: familiarizing with the data, coding, developing initial themes, reviewing themes, defining themes, and writing the results. Research questions of this study (i.e. awareness, understanding, perceived barriers and suggestions for implementation and dissemination) guided the development of themes. Two authors (A.B. and R.A.J.) read the transcripts independently and separately coded the data. Initial independent coding generated 105 codes, which were discussed collaboratively to establish consensus on code definitions and application. Regular discussions were conducted to refine coding consistency. Three iterative rounds of discussions among the research team were conducted to generate final codes, develop themes, and review and refine the thematic structure. Codes were systematically grouped based on their alignment with research questions to construct the final four themes. Data analysis was managed using NVivo software (version 14).

Sample size and adequacy were determined through information power (Malterud et al. 2016) rather than data saturation as saturation is incompatible with our deductive analytical approach (Braun and Clarke 2021). Information power recognizes that sample size requirements vary based on study characteristics. Our study demonstrated high information power through (i) narrow study aim evaluating stakeholder perspectives on specific WHO 24-hour movement guidelines rather than exploratory research of broad phenomenon; (ii) sample specificity with purposive multistakeholder recruitment sampling ensuring representation across socioecological levels; (iii) deductive analytical framework with predetermined themes structured by research questions rather than emerging from inductive coding; (iv) strong dialogue quality through semi-structured interviews averaging 30 minutes for caregivers and teachers, and 50 minutes for professionals’ group; and (v) contextually specific analysis within a clearly defined cultural and geographical context (Mongolia). These characteristics collectively provided sufficient information power with 38 participants across three stakeholder groups. This approach aligns with Braun and Clarke’s (2021) argument that data saturation is conceptually inappropriate for deductive, question-driven thematic analysis, as it assumes an inductive design where themes emerge from coding until redundancy is reached. In our study, themes were constructed to address specific research questions rather than discovered through iterative data collection.

## Results

Four broad themes were identified: (i) awareness and appropriateness, (ii) perceived factors influencing adoption and implementation, (iii) understanding of the guidelines, and 4) Dissemination and implementation strategies. The four themes and supporting quotes are summarized in Table 1.

**Table 1:** Selected quotes illustrating the identified themes from qualitative interviews.

Theme	Subthemes	Selected quotes
1) Awareness and appropriateness	—	‘I've heard that children should go to bed early and reduce screen time, but I haven't heard about these combined guidelines.’ [Caregiver ID023] ‘I hadn't really thought about the specific minutes for physical activity or screen time before. I've heard about recommended sleep times and screen use, but I hadn't paid much attention’. [Teacher ID012]
2) Understanding of the guidelines	—	‘We just hear that screens are bad, but we don't know why or how much is harmful. Knowing that 60 minutes is the limit is helpful’. [Caregiver ID007] "Daily routine’ or ‘healthy lifestyle’ for children sounds better than ‘behaviour’. [Caregiver ID008] ‘Translating it as ‘movement behavior’ might not resonate well with Mongolians. It might be better to use terms like ‘active lifestyle’ or ‘activity level’ to make it more understandable. The term ‘behavior’ might be too broad and not convey the intended meaning clearly. [Health professional ID037] ‘The term ‘screen time’ is tricky. Does it include holding an iPad, watching TV, or just having the TV on in the background? Does it count if they're playing, and a kids’ TV programme is on? We need clarity on what counts as screen time’. [Caregiver ID025] ‘When I think of active movement that are part of the guidelines, I think of exercises and dedicated physical activities. If just running around is considered as physical activity, then we have plenty of active movement at kindergarten’. [Teacher ID020]
3) Perceived factors influencing adoption and implementation	3.1) Competing priorities	‘It's mostly related to our work. For example, I spend a lot of time commuting, so my wife has to manage the kids alone. During that time, they watch TV. The pandemic also contributed to this habit. My wife started a new job, and we had to keep the kids at home. They got used to watching a lot of TV. Even though we try to limit it, it's hard to break the cycle’. [Caregiver ID022] ‘When we stay at home, she gets bored easily, especially since she doesn't have siblings to play with. So, while I'm cooking or doing something else, I need to keep her occupied, which often means screen time’. [Caregiver ID008] ‘We don't do morning exercises every day. We focus more on the Soroban abacus’. [Teacher ID035]. ‘My main criticism is that the Early childhood teacher education programme at the Mongolian State University of Education focuses more on teaching mathematics and language to children, rather than understanding the importance of the daily routine.’ [Teacher ID009] ‘The health system in our country is built with a focus on treatment and hospital care. Instead of relying on treatment after the disease, we should focus more on prevention, working with ourselves, our lifestyle and habits’. [Health professional ID038]
	3.2) Environmental barriers	‘Improving the environment, like having a playground outside, would be good. The neighbourhoods are very cramped. It's difficult to find enough space for children to play. The playgrounds are not always safe or well-maintained. They are often broken or unsafe’. [Caregiver ID018] ‘In winter, it's harder to go outside due to air pollution and cold weather. On very polluted days, we can't go out at all. We usually go out on weekends when my husband is off work, and we can drive somewhere. So, we really have to schedule it, and drive somewhere with purpose of spending time in fresh air. It’s really hard to do that. But if it's too cold, we stay indoors’. [Caregiver ID008] ‘PE class is 30 minutes. We split children because space is small, and they might collide. When one group actively moves, the other group sits down to play games and wait their turn. So, it would be something like 15 minutes. Active movement is quite limited’. [Teacher ID020]
	3.3) Knowledge and capacity gaps	‘Grandmas think kids should eat a lot. They like to keep the kids chubby. It's tough. The kids don't sit and eat properly. They can’t sit for 30 minutes and just eat properly. So, she puts the iPad in front of them to distract them. While they're distracted, she feeds them. They eat while watching something, and the grandma is happy because the kids ate. But this is wrong, it is starting an addiction. They don't eat food as food; they eat while watching something. It's inevitable when grandma is watching them’. [Caregiver ID025] ‘For the active movement, we don't have a dedicated PE teacher. Each kindergarten should have one’. [Caregiver ID014] ‘Children are generally active throughout the day at kindergarten. However, during planned activities and lessons, they do sit down and focus, sometimes using traditional methods like sitting at desks to maintain order and attention’. [Teacher ID026] ‘So, the routine at home is completely different. Unlike us, they don't have a set nap time during the day’. [Teacher ID012] ‘For parents, the main challenge is balancing their busy schedules. While some parents make productive use of weekends by taking their children outdoors, others might not know how to engage their children in active movement at home and end up letting them use mobile phones’. [Teacher ID019] ‘Screen time has become excessive and unregulated. It might get better if he goes to kindergarten.’ [Caregiver ID007]
4) Dissemination and Implementation strategies	—	‘Explaining the negative effects of too much screen time, poor sleep, and lack of physical activity would be useful. People know these are bad, but specific consequences would make it more convincing.’ [Caregiver ID023] ‘If you want to reach all children evenly, you should use Instagram and other platforms together. Otherwise, some children might miss out. It would be good to include everyone. For those who don't use social media or TV, you should visit them and provide information directly. Doing everything using all ways of communication would be effective’. [Caregiver ID017] ‘The teacher spends 7–8 hours a day with my child and is a professional with knowledge. Kindergarten and school teachers are very responsible. So yes, I would trust them’. [Caregiver ID027] ‘If it is integrated into our curriculum, we will follow and implement them’. [Teacher ID035] ‘The main challenge is finding suitable songs and exercises. It takes time to find the right ones and adapt them to the children's age. Having an app with age-appropriate Mongolian songs and exercises would reduce the workload. [Teacher ID010] ‘If we don't properly explain the concept of movement guidelines, people might misunderstand it as children needing to be constantly active. This could overshadow the importance of children learning to sit still and focus which are essential skills for preparing for school. We might end up neglecting these crucial aspects’. [Teacher ID009] ‘Parents often refer to these Pink booklets for information. If we include recommendations, like no screen time for children under 1-year-old, it could help instil these practices in parents’ minds’. [Health professional ID037] ‘Without sufficient promotion and advocacy meetings, the results won't be effectively integrated into sectoral regulations’. [Policy professional ID032]

### Awareness and appropriateness

Participants across all three groups demonstrated consistent responses regarding their awareness of the WHO guidelines and their perceptions about the appropriateness of the guidelines for the Mongolian context. Most participants (90%, *n* = 34) had no prior knowledge of the WHO 24-hour movement guidelines for early childhood. Caregivers were familiar with general health advice that physical activity was beneficial, sleep was important, and screen time should be limited but were unaware of the specific time-based recommendations or the integrated 24-hour approach.

Only one caregiver, one teacher, and two health and policy professionals (10%, *n* = 4) were aware of the guidelines. The caregiver had heard of the guidelines through personal research into child sleep routines, the teacher had encountered the guidelines through professional training, and the health and policy professionals had prior knowledge of the guidelines through their work.

Despite limited awareness, participants across all groups expressed strong support for adopting and implementing the WHO guidelines in Mongolia once they learned about them. Participants consistently suggested that the guidelines would potentially have a positive impact on children's health, provide helpful advice for families and educators, and offer clear, evidence-based recommendations. Across all groups, participants viewed the guidelines as culturally appropriate for the Mongolian context. There was consensus that adopting and implementing the WHO guidelines in Mongolia was necessary and would potentially help strengthen the country's public health policy related to child health.

### Understanding of the guidelines

Across participant groups, there was a general consensus that the WHO 24-hour movement guidelines were clear and understandable and that the daily recommendations for physical activity, sedentary behaviour, and sleep itemized in the guidelines were easy to follow.

An interesting finding was that while caregivers and teachers recognized that ‘screens are bad’, they could not say what were the health consequences of excessive sedentary screen time or specify appropriate time limits. This lack of detailed knowledge appeared to make it difficult for them to make informed decisions about children’s screen use (Table 1, Caregiver ID007). The similar pattern was observed for all components of 24-hour movement behaviours.

Participants suggested several elements that could be modified for the Mongolian context. These suggestions focused on specific terminology used in the guidelines. For example, terms such as ‘high-intensity physical activity’, ‘moderate-to-vigorous-intensity physical activity (MVPA)’, and ‘movement behaviours’ were unfamiliar to many and were considered as too technical for the general public. Participants across all groups were not clear of the meaning of different physical activity intensities (light, moderate, moderate-to-vigorous, and high-intensity) and struggled to match specific physical activities to their corresponding intensity classifications. All participants thought that the term ‘movement behaviours’ was related to movement or physical activity, rather than combination of physical activity, sedentary behaviour and sleep. They suggested that more simple phrases such as ‘healthy lifestyles’ would be better understood within the Mongolian context when referring to the 24-hour movement behaviour concept. Additionally, caregivers suggested that the use of screen time was potentially confusing. They suggested that it was not clear if screen time included using just television, tablets, or mobile phones or all of them, and how to account for background television viewing. Participants also suggested that further clarification was needed between the terms ‘physical activity’ and ‘exercise’. Some of caregivers and teachers misunderstood physical activity as structured exercises and were unsure whether children’s general movement and playtime counted as physical activity (Table 1, Teacher ID020). There were no differences identified in understanding of the guidelines between urban and rural stakeholders.

### Perceived factors influencing adoption and implementation

This theme encompassed three subthemes: competing priorities, environmental barriers, and knowledge and capacity gaps.

#### Competing priorities

Competing priorities were perceived as being a barrier for implementation of the WHO 24-hour movement guidelines in Mongolia. Caregivers consistently identified work and family obligations as primary barriers to supporting their children's adherence to the WHO 24-hour movement guidelines. Work-related barriers included working long hours, weekend shifts, long commutes and both parents working full-time. Family obligations included cooking meals, attending to other children, and doing household chores. Parents described struggling to balance these multiple responsibilities and suggested that movement behaviours were often set aside as they focused more on these perceived urgent responsibilities. The perceived competing barriers suggested by caregivers meant that they often reverted to screen time to occupy their children while they attended to the responsibilities (Table 1, Caregiver ID008).

Teachers suggested that kindergarten schedules were often dominated by school readiness programmes, which focused on literacy, numeracy, or art classes, leaving limited time for active play. While some teachers acknowledged the importance of physical activity for young children and provided morning exercise classes, most did not view it as a priority. Those that did not view physical activity as a priority also mentioned that they had limited knowledge about developmental benefits of physical activity. As a result, in some cases, physical activity time was replaced with seated learning tasks or with optional extracurricular activities such as Soroban abacus class (i.e. Japanese abacus for developing mental math skills).

Teachers and professionals suggested that movement behaviours were not overtly prioritized in the curriculum and policies within the Mongolia’s education and health systems. Within the educational system, cognitive development and school readiness were consistently prioritized over movement behaviours in early childhood programmes (Table 1, Teacher ID009). Within health system, health professionals highlighted that Mongolia’s health system remains primarily treatment-focused, with limited emphasis on prevention and health promotion. This approach means that healthcare resources and infrastructure are predominantly allocated towards managing existing diseases rather than preventing illness before it occurs through health education, early intervention, or addressing the social and environmental health determinants. Participants emphasized that addressing this imbalance would require systemic change, including a stronger commitment to integrating movement behaviours into early childhood development frameworks. They also highlighted the importance of collaboration between the health and education sectors to ensure the guidelines are meaningfully adopted and sustained.

#### Environmental barriers

Participants identified environmental factors as potential barriers to movement guideline implementation, describing how physical and environmental constraints limited children's opportunities for physical activity, resulting in increased sedentary behaviour. Both caregivers and professionals suggested that resources within neighbourhoods, such as the availability of playgrounds and green spaces as main factors for children’s outdoor play. Caregivers living in apartments in Ulaanbaatar specifically cited the lack of proper playgrounds as a major barrier to outdoor play and physical activity. Caregivers had concerns about their children’s safety in the neighbourhoods, suggesting they would allow their children outside more frequently if play areas were enclosed and separated from roads, with appropriate, safe and child-friendly playground equipment. As a result, urban caregivers tended to rely on sedentary and screen-based activities to occupy their children. In contrast, caregivers living in rural provinces and peri-urban areas (i.e. ger districts on the outskirts of the city) suggested that children had greater opportunities for outdoor play, often in yards or nearby natural green spaces.

Teachers identified environmental barriers that limited opportunities for movement and physical activity. Kindergarten teachers from urban area suggested that it was difficult for children to participate in adequate physical activity due to overcrowding, with some small classrooms accommodating up to 40 children (Table 1, Teacher ID020). The small space within the classrooms and the large class sizes meant that children had minimal opportunities for physical activities throughout the day.

All stakeholders noted climate-related barriers for outdoor physical activity, particularly cold weather. During winter months, temperatures drop to −30°C in Ulaanbaatar and reach as low as −40°C in rural Western provinces. However, the most challenging barrier for urban residents of Ulaanbaatar city was the winter air pollution. During these months, air pollution levels reach hazardous levels and exceed the WHO-recommended safe levels by 10-fold (World Health Organization 2019a). Consequently, public health authorities recommend that children spend minimal time outdoors during this period.

#### Knowledge and capacity gaps

Caregivers suggested that their knowledge, beliefs, and attitudes of the grandparents could potentially impact their children's movement behaviours. Within the Mongolian context, many children, particularly those who do not attend kindergarten, are cared for by their grandparents and thus their knowledge, beliefs, and attitudes inform the experiences of the children. Some carers in this study suggested that the grandparents who cared for their children believed that the children were healthy if they ate a lot. Parents also suggested that grandparents often allowed children to watch screens during mealtimes, so they eat enough food. Parents suggested that they felt constrained in addressing the knowledge gap and changing the daily routines and behaviours of children due to their dependency on grandparents for childcare.

Kindergarten teachers identified knowledge and capacity constraints as barriers to children's physical activity. Teachers suggested that the lack of physical education (PE) teachers was a barrier, which limited kindergartens’ perceived ability to provide physical activity programming. Additionally, teachers reported that they did not fully understand the importance of physical activity for young children and were more focused on cognitive tasks. Many teachers believed that children already had sufficient movement when at kindergarten, which created a perception barrier that children may be achieving recommended levels of physical activity.

Teachers identified barriers originating from the home environment, noting that parents’ lifestyle had an impact on children's behaviours. They observed how parental habits and practices, including lack of routine and excessive screen time due to parents’ own lifestyle, habits, and workload, influenced children's movement behaviours and created challenges for implementing healthy daily routines at home. Conversely, instead of establishing healthy daily routines at home, many parents viewed kindergartens as solely responsible for instilling healthy behaviours in their children. They tended to rely on kindergartens to provide structure and daily routines.

### Dissemination and implementation strategies

Participants across all groups proposed a range of strategies to enhance awareness, acceptance, and implementation of the WHO 24-hour movement guidelines within the Mongolian context. Caregivers emphasized the importance of making guidelines accessible. They suggested that messages should clearly explain both the benefits of following the guidelines and the potential negative consequences of not doing so, particularly regarding children’s health and development. All stakeholder groups recommended that the dissemination strategies should use simple language, examples of activities and pictures to illustrate recommended behaviours. Dissemination through multiple sources of information (including television, social media, health professionals, and trusted community figures) was seen as essential to reach families across different settings.

Kindergartens were identified as a central setting for guideline implementation and dissemination. Caregivers viewed teachers as key figures in promoting healthy movement behaviours. They described teachers as trusted, authoritative figures who know their children well and therefore have the right to provide advice, including advice on movement behaviours (Table 1, Caregiver ID027). Teacher–parent meetings were identified as a possible effective way to disseminate the guidelines to caregivers and families.

Teachers themselves recognized this responsibility. They emphasized the importance of including the guidelines in the kindergarten curriculum, so that promoting healthy movement behaviours became a routine part of early childhood education. To support this integration, teachers expressed a need for professional training and resources. Specifically, they requested practical materials and a database of activities easily accessible through mobile or web-based applications. Teachers suggested that these digital resources should include traditional Mongolian games, songs, and dances that were culturally relevant and designed to promote physical activity among children. They noted that without such resources, increasing children's active movement might place additional burden on teachers who must create or source engaging games and activities themselves.

A notable implementation concern was raised by one principal of an urban private kindergarten, who emphasized the need for careful communication when disseminating and implementing the movement behaviour guidelines. Without proper framing, some parents might misinterpret that promoting physical activity to mean that children should always be moving. However, the actual goal is creating an appropriate balance between active and calm learning activities throughout the day. This concern reflects the perceived challenge of encouraging physical activity while ensuring children also develop the skills needed for school readiness. Interestingly, their concern focused on the physical activity component without mentioning other components of the 24-hour movement behaviours.

Health and policy professionals emphasized the need to embed the guidelines into existing systems within the education and health sectors to ensure effectiveness and sustainability. They recommended that the guidelines be incorporated into pre-primary education core curriculum, early childhood teacher training programmes, and health education classes in schools. Another suggestion was to include the guidelines in the ‘Pink Book’, which is a standard national document for monitoring child health and development. This document is provided to all children in Mongolia and serves as a comprehensive health record for tracking development milestones, follow-up examinations and vaccinations until school age. Professionals also called for advocacy campaigns targeting decision-makers to promote the guidelines at the national level and secure policy support for their implementation.

## Discussion

This study examined perceptions of the WHO 24-hour movement guidelines for the early years among Mongolian stakeholders including caregivers of 0- to 5-year-old children (interpersonal level of the SEM), kindergarten teachers, and health professionals (community level of the SEM), and public health researchers (policy/systems level of the SEM). Stakeholders were predominantly unaware of the WHO guidelines, an important finding distinguishing this context from Western and high-income settings where guideline awareness is often strong (Faulkner et al. 2016, Stanley et al. 2020, Capio et al. 2022). Despite consistently low awareness of the WHO guidelines from all stakeholder groups, they demonstrated strong support for adoption and implementation. Stakeholders perceived the guidelines as culturally appropriate and acknowledged their importance for children’s health and development. They identified potential implementation barriers and suggested practical steps for improving future dissemination in the Mongolian context. These findings represent the first examination of stakeholder perspectives on WHO 24-hour movement guidelines in Central Asia. Our findings provide important preliminary insights into health sector and community stakeholder views on potential adoption that can inform multisectoral consultation, including essential engagement with education sector officials.

Few studies have been published exploring WHO guideline adoption in LMICs and none to our knowledge in Central Asian contexts. In contrast to this study, Capio et al. (2022) reported that most early childhood teachers (58.3–86.9%) in Hong Kong were aware of their national movement guidelines ‘Physical Activity Guide’ (adapted from the WHO guidelines). This difference is perhaps not surprising because previous studies examined post-adoption awareness, whereas our study explored awareness prior to adoption (Faulkner et al. 2016, Stanley et al. 2020, Capio et al. 2022, Draper et al. 2022). This awareness gap among caregivers and teachers, who would be the main users of the guidelines in practice represents a notable difference from high-income countries. Stakeholders indicated formal policy and curriculum integration may be necessary foundational steps, as without formal integration, these global guidelines will remain unknown to end users. However, comprehensive implementation planning would require broader policy consultation and warrants further investigation.

Caregivers and teachers demonstrated surface-level familiarity with health-related behaviours (physical activity is beneficial, sleep is important, and screen time should be limited) but lacked deeper understanding that is necessary to translate this knowledge into prioritized action. This knowledge gap could undermine guideline implementation if left unaddressed. Research in physical activity shows that basic knowledge alone (knowing that physical activity is good for health) is not sufficient to influence behaviour, and that more detailed knowledge is needed (Fredriksson et al. 2018 ). Importantly, stakeholders recognized this barrier and suggested specific modifications to enhance understanding and usability. For example, stakeholders found ‘24-hour movement behaviours’ confusing, preferring familiar phrases like ‘healthy lifestyle’ or ‘daily routine’, commonly used in Mongolian when discussing young children’s activities throughout the day. However, this terminology change may lead to a change in its conceptual meaning. For example, ‘healthy lifestyle’ could also include psychological health, oral health and nutrition beyond the WHO guidelines’ movement focus, potentially affecting implementation. Participants suggested education about movement behaviours’ specific scope should be integrated into dissemination strategies, aligning with the WHO’s contextualization guidance (World Health Organization 2023).

While some barriers were consistent with previous studies (e.g. lack of physical spaces, weather and safety) (Hesketh et al. 2017, Capio et al. 2022), others appeared specific to the Mongolian context and represent a novel contribution. Traditional multigenerational childcare arrangements where grandparents play a central caregiving role, reflects Mongolia’s nomadic heritage and cultural values around extended family support (Gan-Yadam 2023). This pattern might also be reinforced by economic factors including limited childcare availability (Nikolova and Polansky 2022). Urban parents rely on grandparents due to work demands, while in rural areas, herding families often send children to live with grandparents or relatives during the school year to access preschool education (Batsaikhan 2025). In our study parents reported generational differences in screen time, feeding and food choices, aligning with research on grandparents’ influence on children’s diet (Young et al. 2018, Marr et al. 2022). These findings suggest that effective dissemination strategies in Mongolia may therefore need to target grandparents alongside parents and teachers through culturally appropriate interventions that respect traditional roles while promoting evidence-based practices.

Competing priorities were identified as a perceived major barrier to guideline compliance across all SEM levels. At the policy level, school readiness and cognitive development are prioritized in the kindergarten curricula over movement behaviours. Consequently, kindergarten teachers in our sample appeared to prioritize classes intended to prepare preschoolers for formal schooling such as literacy, and numeracy, often replacing active play periods while claiming that ‘children need to learn to sit still’. Parents reinforce this pattern by enrolling children in extracurricular activities frequently scheduled during designated nap or active play times, reflecting cultural prioritization of cognitive skills similar to Hong Kong findings (Capio et al. 2022). These practices and constraints suggest that caregivers and teachers have an incomplete understanding of how movement behaviours actually support the developmental outcomes they value. Research demonstrates that compliance with the 24-hour movement guidelines is associated with better socio-cognitive development and school readiness in preschool children (Rollo et al. 2020, Mavilidi et al. 2025). Therefore, dissemination strategies would need to connect movement behaviours to developmental outcomes that parents value, using local examples and visual materials in Mongolian language through parent education sessions, health consultations, and community workshops. These stakeholder-suggested strategies provide a starting point for developing culturally adapted dissemination approaches; however, their effectiveness would need to be evaluated through pilot implementation.

Stakeholders in this study identified kindergartens as potentially optimal implementation setting, given that children spend an average of 8–10 hours daily which represent the majority of their wake time. This aligns with previous research demonstrating kindergartens (i.e. early childhood education centres) as an ideal setting for promoting movement behaviour and in particular the physical activity component of the guidelines (Jones et al. 2019). Kindergarten-based implementation is especially critical in urban areas where hazardous winter air pollution makes outdoor activity unsafe for much of the year, necessitating year-round indoor infrastructure.

Caregivers in our sample acknowledged teachers as trusted figures while teachers demonstrated willingness to accept this responsibility, creating mutual support between stakeholders. Parent-teacher collaboration is widely recommended in the literature as a way to increase effectiveness of health interventions (Verjans-Janssen et al. 2018). However, caregivers in our study appeared to delegate responsibility entirely to educational institutions rather than collaborating with teachers. Participants also identified barriers to effective collaboration, including teachers’ time constraints, resource limitations, and varying confidence levels to support the implementation of the guidelines. Stakeholders suggested that addressing these barriers would require a policy-level support from both health and education sectors. Moreover, education ministry consultation would be essential to determine the feasibility of curriculum integration, professional development, and development and allocation of necessary resources including comprehensive database of activities and games to promote healthy movement behaviours.

Health and public health professionals in our sample suggested leveraging existing kindergarten infrastructure and health policy frameworks rather than developing new systems, which is particularly important for a LMIC with limited resources This approach aligns with implementation science evidence, showing that interventions integrated within established systems achieve greater success and sustainability than those requiring new frameworks (Peters et al. 2020, Nathan et al. 2023). For Mongolia, this approach may be especially relevant given the public health priority of addressing the burden of NCD that account for approximately 74% of deaths nationally (World Health Organization 2025). Since early prevention of non-communicable diseases requires establishing healthy behaviours in childhood, stakeholder support for guideline implementation could contribute to broader NCD prevention efforts (Okely et al. 2018, Yan and Mi 2021).

### Strengths and limitations

The strength of this study is the recruitment of participants from multiple levels of the SEM, which provided diverse perspectives on children’s movement behaviours. This multilevel approach captured insights from caregivers and teachers as well as health sector system-level stakeholders, providing a more complete understanding than single-stakeholder studies.

Several limitations should be acknowledged. First, policy representation was limited to health sector (*n* = 5) without education, sport or urban planning officials, which are critical for comprehensive implementation. Our findings represent health sector and community perspectives, not multisectoral assessment or definitive implementation guidance. Second, kindergarten teachers and principals provided insights into barriers and facilitators within the education sector. These do not represent policy-level perspectives and cannot substitute for education ministry consultation. Third, while our focus on caregivers of children 0–5 years captured real-time perspectives, future research could valuably incorporate retrospective insights from caregivers of older children, who may offer valuable reflections on early movement behaviours and their developmental impacts. Finally, implementation feasibility, resource requirements and multisectoral coordination require assessment beyond the scope of this study. Future research engaging education policymakers and other sectors is essential to validate our preliminary findings and determine implementation feasibility.

## Conclusion

This study provides the first examination of stakeholder perspectives on WHO 24-hour movement guidelines in Mongolia. Our novel contributions include identifying implementation challenges that differ from Western contexts (e.g. lack of awareness, environmental barriers requiring different strategies and culturally specific caregiving patterns). These preliminary insights suggest that successful guideline adoption may require culturally adapted and kindergarten-centred strategies addressing knowledge gaps and systemic barriers. Our findings provide a foundation for evidence-informed multisectoral dialogue about guideline adaptation in Mongolia and offer insights for similar Central Asian and postsocialist countries.

## Supplementary Material

czag037_Supplementary_Data

## Data Availability

The qualitative data underlying this article cannot be shared publicly to protect privacy and confidentiality of participants. Deidentified data may be shared on reasonable request to the corresponding author.

## Reflexivity statement

The authors include two females and one male and span multiple levels of seniority, from PhD candidate to established professors. The lead author (A.B.) is a Mongolian national working as a research officer at the National Centre for Public Health of Mongolia while pursuing doctoral studies in Australia, bringing knowledge of Mongolia's public health system and cultural context. The two Australian-based supervisors (R.A.J. and A.D.O.) specialize in early childhood health and physical activity research. The lead author's position at the National Centre for Public Health facilitated access to diverse stakeholders and enabled data collection in Mongolian, the participants’ native language.
