# Supplementary material for: WHO guidelines on physical activity, sedentary behaviour, and sleep for children under 5: a qualitative study of Mongolian stakeholder perceptions
Source: Health Policy Plan. 2026 Mar 17;41(5):809–20. doi: 10.1093/heapol/czag037 (PMC13187624; doi:10.1093/heapol/czag037)
Supplement: czag037_Supplementary_Data [file czag037_supplementary_data.zip › Supplementary File 2. Interview guides.docx]

**Supplementary File 2**

**INTERVIEW GUIDE with caregivers**

| **Section** | **Questions** |
| --- | --- |
| **Introduction** | - Can you tell me briefly about yourself? How old is your child? - Can you describe your child’s typical daily routine, including sleep, physical activity and screen time? |
| **Main part** | Awareness about the WHO guidelines:   - Before this study, were you aware of the WHO guidelines on 24-hour movement behaviours? If yes, how did you first hear about them? - Have you heard of the term “movement behaviours”? If yes, what do you think it means? |
|  | Understanding *(translated guidelines will be provided):*   - What are your thoughts on these guidelines that includes recommendations on how much time young children should spend being physically active, sleeping, using screens, and sitting for long periods? - Do you find the wording of the guidelines easy to understand? How would you rate the language used in terms of clarity? - Do you think the guidelines are culturally appropriate for Mongolia? |
|  | Perceived benefits:   - How would you feel about following these guidelines if they were introduced in Mongolia? - What impact, if any, do you think following these guidelines could have on your child’s health and development? - Do you think these guidelines could help reduce any concerns you have about your child’s physical activity, sleep, or screen time? Why or why not? |
|  | Perceived barriers:   - Can you think of any specific challenges that might make it hard for children to follow the recommendations for physical activity? For sleep? For screen time? For prolonged restrained sitting time? - Which part of the guidelines do you find especially difficult to follow for your child? |
| **Suggestions** | Suggestions for improving language of the guidelines:   - What changes would you suggest to make these guidelines more useful and practical for parents and children in Mongolia? - Are there any specific words or terms that you think should be changed to make the guidelines easier to understand and use? |
|  | Suggestions for dissemination and implementation:   - Who would be the best individual/organization/media to provide information about the guidelines to you? - What kind of support or resources do you think would help you follow the guidelines and manage your child’s physical activity, sleep, and screen time better? |
| **Conclusion** | - Do you have any additional comments or suggestions regarding the guidelines? - Is there anything else you would like to share that we have not covered? |

**INTERVIEW GUIDE with teachers**

| **Section** | **Questions** |
| --- | --- |
| **Introduction** | - Can you tell me briefly about yourself? How long have you been working in the kindergarten? - How do you incorporate physical activity, sleep, and screen time management into the daily schedule of your classroom? |
| **Main part** | Awareness about the WHO guidelines:   - Before this study, were you aware of the WHO guidelines on 24-hour movement behaviours? If yes, how did you first hear about them? - Have you heard of the term “movement behaviours”? If yes, what do you think it means? |
|  | Understanding *(translated guidelines will be provided)*:   - What are your thoughts on these guidelines that includes recommendations on how much time young children should spend being physically active, sleeping, using screens, and sitting for long periods? - Do you find the wording of the guidelines easy to understand? How would you rate the language used in terms of clarity? - Do you think the guidelines are culturally appropriate for Mongolia? |
|  | Perceived benefits:   - How important do you believe it is for preschool children to follow these guidelines? - Do you believe these guidelines would be useful if they were included in education curricula in Mongolia? Why or why not? - How might these guidelines affect your role as a teacher in promoting healthy behaviours among children? |
|  | Perceived barriers:   - Can you think of any specific challenges that might make it hard for children to follow the recommendations for physical activity? For sleep? For screen time? For prolonged restrained sitting time? - Based on your experience, what factors might make it difficult for teachers or kindergartens to implement these guidelines effectively? |
| **Suggestions** | Suggestions for improving language of the guidelines:   - What changes would you suggest to make these guidelines more useful and practical for parents and children in Mongolia? - Are there any specific words or terms that you think should be changed to make the guidelines easier to understand and use? |
|  | Suggestions for dissemination and implementation:   - Who would be the best individual/organization/media to provide information about the guidelines to you? - How well do the current education curricula align with the WHO guidelines? - What additional support would you need to better implement these guidelines in your classroom? - What do you think are the key considerations if we want parents to be motivated to help their children meet the movement guidelines? |
| **Conclusion** | - Do you have any additional comments or suggestions regarding the guidelines? - Is there anything else you would like to share that we have not covered? |

**INTERVIEW GUIDE with policy professionals**

| **Section** | **Questions** |  |
| --- | --- | --- |
| **Introduction** | - Can you tell me about your occupation and how it relates to children’s health? - How long have you been involved in this field? |  |
| **Main part** | Awareness about the WHO guidelines:   - Before this study, were you aware of the WHO guidelines on 24-hour movement behaviours? If yes, how did you first hear about them? - Are you familiar with the concept “movement behaviours”? If yes, what are they representing? |  |
|  | Understanding *(translated guidelines will be provided):*   - What are your thoughts on integrated guidelines that includes recommendations for physical activity, sleep, and sedentary behaviour? - Do you find the wording of the guidelines easy to understand? How would you rate the language used in terms of clarity? - Do you think the guidelines are culturally appropriate for Mongolia? |  |
|  | Perceived benefits:   - Do you think these integrated guidelines useful if adopted in Mongolia? - What opportunities do you see for improving health policy alignment with the WHO guidelines? - How can public health research inform and shape policy decisions regarding children's movement behaviours? |  |
|  | Perceived barriers:   - Can you describe any specific barriers that could prevent children from adhering to these guidelines? - What are the biggest challenges in implementing and enforcing policies related to physical activity and other health-related behaviours? - What challenges do you face in ensuring that research findings are effectively communicated and implemented in public health initiatives? |  |
| **Suggestions** | Suggestion for improvement:   - What changes would you suggest to make these guidelines more relevant and practical for the Mongolian context? - Are there any specific words or terms that you think should be replaced to improve their understanding and implementation? |  |
|  | Suggestion for dissemination and implementation:   - Who would be the best individual/organization/media to provide information about the guidelines to the population? - What strategies would be effective in promoting and implementing the WHO guidelines at the community or national level in Mongolia? |  |
| **Conclusion** | - Do you have any additional comments or suggestions regarding the guidelines? - Is there anything else you would like to share that we have not covered? | |

**INTERVIEW GUIDE with health professionals**

| **Section** | **Questions** |
| --- | --- |
| **Introduction** | - Can you tell me about your occupation? - How long have you been involved in this field? |
| **Main part** | Awareness about the WHO guidelines:   - Before this study, were you aware of the WHO guidelines on 24-hour movement behaviours? If yes, how did you first hear about them? - Are you familiar with the concept “movement behaviours”? If yes, what are they representing? - What health outcomes do you observe in young children that are related to their physical activity, screen time, and sleep patterns? - Do you assess or monitor these behaviours in your practice?" |
|  | Understanding *(translated guidelines will be provided):*   - What are your thoughts on integrated guidelines that includes recommendations for physical activity, sleep, and sedentary behaviour? - Do you find the wording of the guidelines easy to understand? How would you rate the language used in terms of clarity? - Do you think the guidelines are culturally appropriate for Mongolia? |
|  | Perceived benefits:   - How important is it for young children to follow these guidelines, and what health benefits do you anticipate? - Would these guidelines be useful if adopted in Mongolia, and how might they enhance public health strategies? - Would you incorporate these guidelines into your practice when advising parents, and what potential benefits do you see? |
|  | Perceived barriers:   - Based on your experience, what local factors might hinder the implementation of these guidelines for young children? - What challenges might health professionals face in promoting adherence to these guidelines among children? - What barriers do you foresee in integrating these guidelines into existing public health programs or practices? |
| **Suggestions** | Suggestion for improvement:   - What changes would you suggest to make these guidelines more relevant and practical for the Mongolian context? - Are there any specific words or terms that you think should be replaced to improve their understanding and implementation? |
|  | Suggestion for dissemination and implementation:   - Who would be the best individual/organization/media to provide information about the guidelines to the population? - How do you collaborate with parents, educators, or other people to promote healthy movement behaviours in young children? - What role do you see health professionals playing in the implementation of these guidelines? |
| **Conclusion** | - Do you have any additional comments or suggestions regarding the guidelines? - Is there anything else you would like to share that we have not covered? |
