# Supplementary material for: WHO guidelines on physical activity, sedentary behaviour, and sleep for children under 5: a qualitative study of Mongolian stakeholder perceptions
Source: Health Policy Plan. 2026 Mar 17;41(5):809–20. doi: 10.1093/heapol/czag037 (PMC13187624; doi:10.1093/heapol/czag037)
Supplement: czag037_Supplementary_Data [file czag037_supplementary_data.zip › Supplementary File 3. COREQ 32-item checklist.docx]

**Supplementary File 3. COREQ 32-item checklist for manuscript titled:**

**"Mongolian stakeholders’ perceptions of WHO Guidelines on Physical activity, Sedentary behaviour and Sleep for children under 5: a qualitative study."**

| **No.** | **Item** | **Description** | **Reported on section and page number** | |
| --- | --- | --- | --- | --- |
| **DOMAIN 1: RESEARCH TEAM AND REFLEXIVITY** | | | |  |
| ***Personal Characteristics*** | | | |  |
| 1 | **Interviewer/facilitator** | Which author/s conducted the interview or focus group? | Methods  Data collection, Page 10 | |
| 2 | **Credentials** | What were the researcher's credentials? E.g. PhD, MD | Methods  Reflexivity, Page 6 | |
| 3 | **Occupation** | What was their occupation at the time of the study? | Methods  Reflexivity, Page 6 | |
| 4 | **Gender** | Was the researcher male or female? | Methods  Reflexivity, Page 6 | |
| 5 | **Experience and training** | What experience or training did the researcher have? | Methods  Reflexivity, Page 6 | |
| ***Relationship with participants*** | | | |  |
| 6 | **Relationship established** | Was a relationship established prior to study commencement? | Methods  Design and sampling,  Page 8 | |
| 7 | **Participant knowledge of the interviewer** | What did the participants know about the researcher? e.g. personal goals, reasons for doing the research | Methods  Reflexivity, Page 7 | |
| 8 | **Interviewer characteristics** | What characteristics were reported about the interviewer/facilitator? e.g. Bias, assumptions, reasons and interests in the research topic | Methods  Reflexivity, Page 6 | |
| **DOMAIN 2: STUDY DESIGN** | | | |  |
| ***Theoretical framework*** | | | |  |
| 9 | **Methodological orientation and Theory** | What methodological orientation was stated to underpin the study? e.g. grounded theory, discourse analysis, ethnography, phenomenology, content analysis | Methods  Data analysis, Page 11-12 | |
| ***Participant selection*** | | | |  |
| 10 | **Sampling** | How were participants selected? e.g. purposive, convenience, consecutive, snowball | Methods  Design and sampling,  Page 7-8 | |
| 11 | **Method of approach** | How were participants approached? e.g. face-to-face, telephone, mail, email | Methods  Design and sampling,  Page 8 | |
| 12 | **Sample size** | How many participants were in the study? | Methods  Design and sampling,  page 8 | |
| 13 | **Non-participation** | How many people refused to participate or dropped out? Reasons? | NA | |
| ***Setting*** | | | |  |
| 14 | **Setting of data collection** | Where was the data collected? e.g. home, clinic, workplace | Methods  Data collection, Page 10 | |
| 15 | **Presence of non-participants** | Was anyone else present besides the participants and researchers? | NA | |
| 16 | **Description of sample** | What are the important characteristics of the sample? e.g. demographic data, date | Methods  Design and sampling,  page 8-9 | |
| ***Data collection*** | | | |  |
| 17 | **Interview guide** | Were questions, prompts, guides provided by the authors? Was it pilot tested? | Methods  Data collection, Page 10 | |
| 18 | **Repeat interviews** | Were repeat interviews carried out? If yes, how many? | NA | |
| 19 | **Audio/visual recording** | Did the research use audio or visual recording to collect the data? | Methods  Data recording and translation, Page 11 | |
| 20 | **Field notes** | Were field notes made during and/or after the interview or focus group? | Methods  Reflexivity, Page 7 | |
| 21 | **Duration** | What was the duration of the interviews or focus group? | Methods  Data collection, Page 10 | |
| 22 | **Data saturation** | Was data saturation discussed? | Methods  Data analysis, Page 12 | |
| 23 | **Transcripts returned** | Were transcripts returned to participants for comment and/or correction? | Methods  Data recording and translation, Page 11 | |
| **DOMAIN 3: ANALYSIS AND FINDINGS** | | | |  |
| ***Data analysis*** | | | |  |
| 24 | **Number of data coders** | How many data coders coded the data? | Methods  Data analysis, Page 12 | |
| 25 | **Description of the coding tree** | Did authors provide a description of the coding tree? | NA | |
| 26 | **Derivation of themes** | Were themes identified in advance or derived from the data? | Methods  Data analysis, Page 11-12 | |
| 27 | **Software** | What software, if applicable, was used to manage the data? | Methods  Data analysis, Page 12 | |
| 28 | **Participant checking** | Did participants provide feedback on the findings? | NA | |
| ***Reporting*** | | | |  |
| 29 | **Quotations presented** | Were participant quotations presented to illustrate the themes / findings? Was each quotation identified? e.g. participant number | Table 1. Quotes | |
| 30 | **Data and findings consistent** | Was there consistency between the data presented and the findings? | Results, Page 13-21 | |
| 31 | **Clarity of major themes** | Were major themes clearly presented in the findings? | Results, Page 13-21 | |
| 32 | **Clarity of minor themes** | Is there a description of diverse cases or discussion of minor themes? | NA | |
