# Supplementary figures and images for: WHO guidelines on physical activity, sedentary behaviour, and sleep for children under 5: a qualitative study of Mongolian stakeholder perceptions
Source: Health Policy Plan. 2026 Mar 17;41(5):809–20. doi: 10.1093/heapol/czag037 (PMC13187624; doi:10.1093/heapol/czag037)

**Supplementary File 1. Mongolian-translated version of the WHO guidelines**


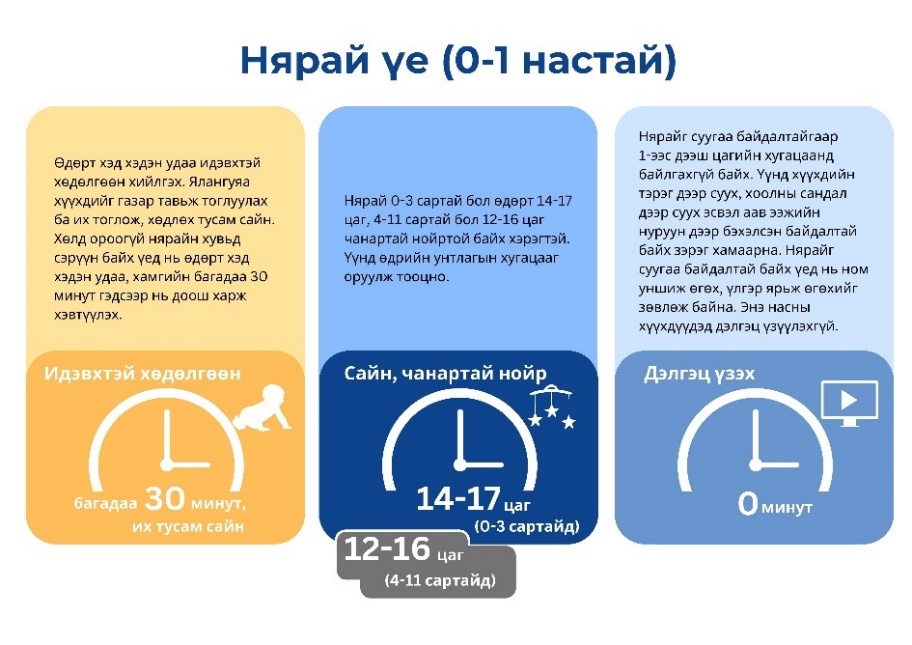

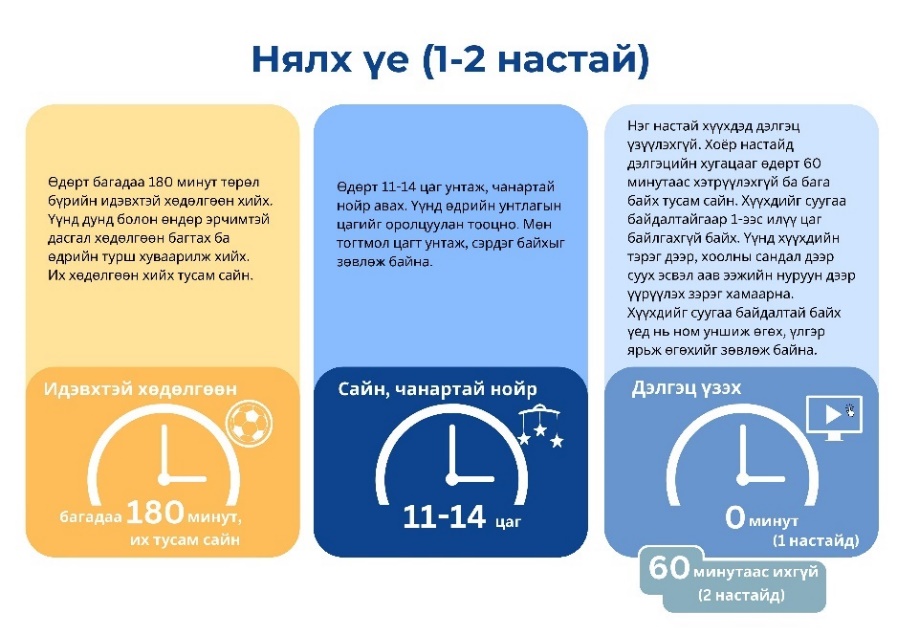

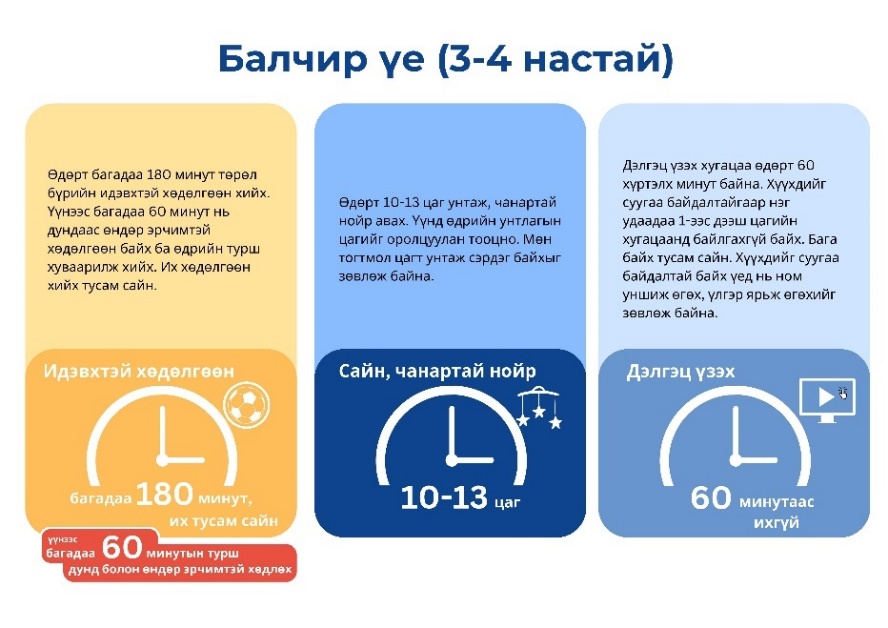

Supplement: czag037_Supplementary_Data [file czag037_supplementary_data.zip › Supplementary File 1. Mongolian version of the guidelines.docx]
